# Supplementary material for: Epistatic contributions to human traits via transcription factor mechanisms
Source: medRxiv. 2025 Sep 29:2025.09.28.25336826. Preprint. [Version 1] doi: 10.1101/2025.09.28.25336826 (PMC12622120; doi:10.1101/2025.09.28.25336826)
Supplement: Supplement 2 [file media-2.pdf]

A Tables

Nuclear Hormone Receptor	Gene Symbol	# bQTLs	# fQTLs	Hormone	Protein
Androgen receptor	AR	8	14 hQTLs; 21 globQTLs	Testosterone	Sex Hormone Binding Globulin
Progesterone receptor	PGR	1	9 hQTLs; 32 eQTLs	Progesterone	
Estrogen receptor	ESR1	77	6 hQTLs; 21 globQTLs; 4 eQTLs	Estradiol	Sex Hormone Binding Globulin
Glucocorticoid receptor	NR3C1	10	2 hQTLs; 11 eQTLs	Cortisol	
Vitamin D receptor	VDR	12	8 hQTLs; 2 eQTLs	25-hydroxyvitamin D	
Nuclear receptor subfamily 1	NR2F1	2	16 eQTLs		
Nuclear receptor subfamily 2	NR2F2	1	3 eQTLs		
Hepatocyte nuclear factor 4α	HNF4A	3	5 eQTLs		
Hepatocyte nuclear factor 4γ	HNF4G	5	7 eQTLs		

Table 1: **Summary of the QTLs studied for this manuscript.** For each of 9 NHRs, we report the number of high-quality bQTLs, as well as fQTLs whose mechanism is either by altered mRNA expression of the TF (eQTLs), altered ligand abundance directly by the hormone (hQTLs), or by Sex hormone binding globulin (globQTLs). Additional information on each SNP can be found in **Supplemental Table S1 and S2**.

Table 2: All replicated 2-point interactions across 6 NHRs

bQTL	Outcome	Transcription factor	# Trans-actors
rs2553234	M86-M90 Other osteopathies	AR	2
rs8180759	N93 Other abnormal uterine and vaginal bleeding	AR	2
rs4977574	Poultry intake	AR	2
rs12626817	I26-I28 Pulmonary heart disease and diseases of pulmonary circulation	PGR	2
rs12626817	Part of a multiple birth	PGR	2
rs12626817	angina	PGR	2
rs12626817	M70-M79 Other soft tissue disorders	PGR	2

Continued on next page

Table 2 – continued from previous page

bQTL	Outcome	Transcription Factor	# Trans-actors
rs12626817	B95 Streptococcus and staphylococcus as the cause of diseases classified to other chapters	PGR	2
rs12626817	osteopenia	PGR	2
rs12626817	J92 Pleural plaque	PGR	2
rs12626817	cervical problem	PGR	3
rs12626817	J34 Other disorders of nose and nasal sinuses	PGR	3
rs12626817	gall bladder disease	PGR	9
rs12447081	transient ischaemic attack (tia)	ESR1	2
rs17708638	Arm fat mass (left)	ESR1	3
rs17708638	Arm fat mass (right)	ESR1	3
rs6470263	O80 Single spontaneous delivery	HNF4A	2
rs6470263	hayfever/allergic rhinitis	HNF4A	2
rs514795	H25-H28 Disorders of lens	HNF4A	2
rs514795	H25 Senile cataract	HNF4A	2
rs514795	eye trauma	HNF4A	2
rs6470263	uterine problem	HNF4A	2
rs1191818	haematology	HNF4A	2
rs6470263	J35 Chronic diseases of tonsils and adenoids	HNF4A	3
rs1191818	anaemia	HNF4A	3
rs568557	clotting disorder/excessive bleeding	HNF4G	2
rs514795	I89 Other non-infective disorders of lymphatic vessels and lymph nodes	HNF4G	2
rs568557	osteopenia	HNF4G	2
rs568557	heart arrhythmia	HNF4G	2
rs514795	G56 Mononeuropathies of upper limb	HNF4G	3
rs568557	M07 Psoriatic and enteropathic arthropathies	HNF4G	3
rs6470263	L57 Skin changes due to chronic exposure to nonionising radiation	NR2F1	2
rs11642445	K55-K64 Other diseases of intestines	NR2F1	2
rs11642445	neck problem/injury	NR2F1	2
rs6470263	Lymphocyte percentage	NR2F1	2
rs11642445	K04 Diseases of pulp and periapical tissues	NR2F1	2
rs6470263	viral infection	NR2F1	2
rs11642445	Hot drink temperature	NR2F1	2
rs6470263	E20-E35 Disorders of other endocrine glands	NR2F1	3
rs6470263	N32 Other disorders of bladder	NR2F1	3
rs6470263	L55-L59 Radiation-related disorders of the skin and subcutaneous tissue	NR2F1	3
rs6470263	Coffee intake	NR2F1	3
rs11642445	M77 Other enthesopathies	NR2F1	3
rs6470263	chronic obstructive airways disease/copd	NR2F1	4
rs6470263	J40-J47 Chronic lower respiratory diseases	NR2F1	4
rs6470263	F00-F09 Organic, including symptomatic mental disorders	NR2F1	4
rs6470263	M77 Other enthesopathies	NR2F1	4
rs6470263	F05 Delirium, not induced by alcohol and other psychoactive substances	NR2F1	5
rs11642445	Immature reticulocyte fraction	NR2F1	5
rs6470263	K42 Umbilical hernia	NR2F1	6

Continued on next page

Table 2 – continued from previous page

bQTL	Outcome	Transcription Factor	# Trans-actors
rs11642445	K31 Other diseases of stomach and duodenum	NR2F1	7
rs11642445	muscle or soft tissue injuries	NR2F1	8
rs998384	High light scatter reticulocyte count	NR2F2	2
rs998384	Leg fat percentage (left)	NR2F2	2
rs998384	L30 Other dermatitis	NR2F2	2
rs998384	Mean platelet (thrombocyte) volume	NR2F2	2
rs998384	G40 Epilepsy	NR2F2	2
rs998384	Beef intake	NR2F2	3

Table 3: All replicated 3-point interactions across 2 NHRs

bQTL	Outcome	Transcription factor	# Trans-actors
rs4977574	Variation in diet	AR	2
rs12626817	K56 Paralytic ileus and intestinal obstruction without hernia	PGR	2
rs12626817	G47 Sleep disorders	PGR	2
rs12626817	B97 Viral agents as the cause of diseases classified to other chapters	PGR	2
rs12626817	Monocyte count	PGR	3
rs12626817	C15-C26 Malignant neoplasms of digestive organs	PGR	3
rs12626817	L30 Other dermatitis	PGR	3
rs12626817	L20-L30 Dermatitis and eczema	PGR	3
rs2286519	Mean reticulocyte volume	ESR1	2
rs1881561	I60-I69 Cerebrovascular diseases	ESR1	3
